# Silencing of Dicer enhances dacarbazine resistance in melanoma cells by inhibiting ADSL expression

**DOI:** 10.18632/aging.205207

**Published:** 2023-11-15

**Authors:** Yu-Wen Yeh, Tung-Wei Hsu, Yen-Hao Su, Chih-Hsin Wang, Po-Hsiang Liao, Ching-Feng Chiu, Po-Chen Tseng, Tim-Mo Chen, Woan-Ruoh Lee, Yuan-Sheng Tzeng

**Affiliations:** 1 Graduate Institute of Medical Sciences, National Defense Medical Center, Taipei 114, Taiwan; 2 Division of Dermatology, Tri-Service General Hospital Songshan Branch, National Defense Medical Center, Taipei 105, Taiwan; 3 Graduate Institute of Medical Sciences, College of Medicine, Taipei Medical University, Taipei 110, Taiwan; 4 Department of Surgery, Division of General Surgery, Shuang Ho Hospital, Taipei Medical University, Taipei 235, Taiwan; 5 Department of General Surgery, School of Medicine, College of Medicine, Taipei Medical University, Taipei 110, Taiwan; 6 TMU Research Center of Cancer Translational Medicine, Taipei Medical University, Taipei 110, Taiwan; 7 Department of Surgery, Division of Plastic and Reconstructive Surgery, Tri-Service General Hospital, National Defense Medical Center, Taipei 114, Taiwan; 8 Graduate Institute of Metabolism and Obesity Sciences, College of Nutrition, Taipei Medical University, Taipei 110, Taiwan; 9 Department of Ophthalmology, Taipei City Hospital, Renai Branch, Taipei 106, Taiwan; 10 Department of Ophthalmology, School of Medicine, College of Medicine, Taipei Medical University, Taipei 110, Taiwan; 11 Department of Surgery, Zuoying Branch of Kaohsiung Armed Forces General Hospital, Kaohsiung 813, Taiwan

**Keywords:** melanoma, dacarbazine (DTIC), Dicer, adenylosuccinate lyase (ADSL)

## Abstract

Dacarbazine (DTIC) is the primary first-line treatment for advanced-stage metastatic melanoma; thus, DTIC resistance is poses a major challenge. Therefore, investigating the mechanism underlying DTIC resistance must be investigated. Dicer, a type III cytoplasmic endoribonuclease, plays a pivotal role in the maturation of miRNAs. Aberrant Dicer expression may contribute to tumor progression, clinical aggressiveness, and poor prognosis in various tumors. Dicer inhibition led to a reduction in DTIC sensitivity and an augmentation in stemness in melanoma cells. Clinical analyses indicated a low Dicer expression level as a predictor of poor prognosis factor. Metabolic alterations in tumor cells may interfere with drug response. Adenylosuccinate lyase (ADSL) is a crucial enzyme in the purine metabolism pathway. An imbalance in ADSL may interfere with the therapeutic efficacy of drugs. We discovered that DTIC treatment enhanced ADSL expression and that Dicer silencing significantly reduced ADSL expression in melanoma cells. Furthermore, ADSL overexpression reversed Dicer silencing induced DTIC resistance and cancer stemness. These findings indicate that Dicer-mediated ADSL regulation influences DTIC sensitivity and stemness in melanoma cells.

## INTRODUCTION

Melanoma is an extremely aggressive form of skin cancer, accounting for approximately 80% of skin cancer [1–3]. Melanoma treatment depends on the clinical and pathological staging at the time of diagnosis and includes a range of interventions such as surgery, radiation therapy, chemotherapy and combination therapy [4]. Despite the potential for a complete cure through surgical excision in cases of early-stage localized melanoma, approximately 20% of patients progress to advanced-stage metastatic melanoma because of the highly invasive nature of melanoma, which enables rapid dissemination to distant organs [5, 6]. The only chemotherapeutic agent approved by the US Food and Drug Administration for the treatment of inoperable advanced-stage metastatic melanoma is dacarbazine (5-(3,3-dimethyl-1-triazeno)-imidazole-4-carboxamide; DTIC) [7]. However, only 5%–10% of all patients with metastatic melanoma respond to DTIC, and a vast majority of patients experience relapse within a few weeks or months. Furthermore, the rate of 5-year survival for patients with metastatic melanoma is less than 30% [8, 9]. Therefore, elucidating the molecular regulatory mechanisms underlying DTIC resistance and identifying novel molecules for use in melanoma diagnosis, prognosis, prevention, and therapy is vital.

The components of the miRNA biogenesis machinery are inextricably linked to cancer progression [10]. Dicer is critical in controlling miRNA biogenesis and is also involved in chromatin structure remodeling, inflammation, and DNA degradation [11]. Dicer, categorized as a cytoplasmic endonuclease belonging to the RNase III family and is crucial for miRNA maturation [12]. Dysregulation of Dicer expression or activity has been implicated in tumorigenic alterations and is associated with a worsened prognosis in different types of cancer [13]. However, the regulation of Dicer in clinical chemotherapy is a complex and sometimes contradictory process. Research has indicated that low Dicer expression is strongly interrelated with poor survival outcomes in hepatocellular carcinoma (HCC) [14], breast cancer [15], and cervical cancer [16] and that Dicer is involved in the regulation of chemotherapy sensitivity chemotherapy. For instance, Dicer knockdown in breast cancer cells has been demonstrated to induce significant cell cycle arrest and enhances cisplatin sensitivity, possibly through the modulation of particular miRNAs [17]. Dicer impairment has been associated with oxaliplatin resistance and increased metastasis in colon cancers [18]. In addition, the zeste homolog 2 enhancer was demonstrated to regulate Dicer expression, thereby influencing the aggressiveness and chemoresistance of ovarian cancer. [19]. Nevertheless, the specific involvement of in mediating DTIC resistance in melanoma cells is not fully understood.

Accumulating evidence indicates a relationship between the dysregulation of cellular metabolism and the development of cancer drug resistance [20, 21]. Purine metabolism is the primary source of purine nucleotides, which are needed for synthesizing DNA and RNA and provide energy and cofactors necessary for supporting the cancer cell survival and proliferation of cancer cells [22]. Adenylosuccinate lyase (ADSL) is an enzyme required for *de novo* purine biosynthesis. Although ADSL is closely related to the progression of various cancers [23, 24], the exact molecular mechanisms underlying the expression of Dicer and ADSL remain elusive. Consequently, investigation of the relationship between Dicer-mediated ADSL dysregulation and DTIC resistance in melanoma is imperative.

In this study, we investigated the mechanism through which Dicer regulates the therapeutic efficacy of DTIC in melanoma cells. The results revealed that the silencing of Dicer expression led to a substantial increase in the DTIC resistance of melanoma cells, and low Dicer expression level was found to be associated with unfavorable survival outcomes in melanoma patients. To the best of our knowledge, this is the first study to reveal that Dicer regulates ADSL expression and that this regulation is influences the DTIC sensitivity of melanoma cells. Our findings may guide the identification of novel therapeutic targets and biomarkers relevant in melanoma.

## MATERIALS AND METHODS

### Cell cultures

Two human melanoma cell lines, A375 and A2508, were obtained from the American Type Culture Collection (Manassas, VA, USA). These cells were cultured in Dulbecco’s Modified Eagle Medium (Corning Inc., Corning, NY, USA) supplemented with 10% heat-inactivated fetal bovine serum and 1% P/S/A in a humidified incubator at 37°C under 5% CO_2_.

### Cell viability assay

Cell viability was assessed using the 3-(4,5-dimethylthiazol-2-yl)-2,5-diphenyltetrazolium bromide (MTT) assay. MTT powder was dissolved in phosphate-buffered saline to prepare the MTT solution (5 mg/mL). In total, 2 × 10^3^ cells were seeded onto 96-well plates containing the aforementioned complete medium. After 48 and 72 h, the MTT solution was added to the wells and the plates were incubated at 37°C for 3 hr. After incubation, dimethyl sulfoxide was added to each well, = the optical density of the samples was measured at a wavelength of 570 nm.

### Quantitative reverse-transcription polymerase chain reaction (qRT-PCR) assay

Total RNA was extracted from the human melanoma cells using an RNA isolation kit (RNeasy Mini Kit, Qiagen, Venlo, The Netherlands), and cDNA was reverse-transcribed using a Fermentas Reverse Transcriptase Kit (Thermo Fisher Scientific, Waltham, MA, USA) according to the manufacturer’s instructions. qRT-PCR was performed using a qScript One-Step QRT-PCR Kit (Pro-Tech, Taipei, Taiwan). To ensure robust and reliable results, the qRT-PCR experiments were performed at least three times, with each experiment performed in triplicate independent experiments using the CFX96 Touch Real-Time PCR Detection System (Bio-Rad, Hercules, CA, USA). The GAPDH gene was used as an internal control. The specific primers used in the analyses are shown in the Table 1.

**Table 1:** Primer sequences information for qRT-PCR.

**Dicer**	Forward: CTTTCAGTGAGCTGTGCTGC
	Reverse: CCAAAATCGCATCTCCCAGG
**ADSL**	Forward: TAAATTCCGGACATGGCGGC
	Reverse: GTGCAGGCTGGAAATGTGTG
**Oct-4**	Forward: GCAATTTGCCAAGCTCCTGAA
	Reverse: GCAGATGGTCGTTTGGCTGA
**Nanog**	Forward: AATGGTGTGACGCAGGGATG
	Reverse: TGCACCAGGTCTGAGTGTTC
**SOX2**	Forward: GGGGGAATGGACCTTGTATAG
**KLF4**	Forward: GGGAGAAGACACTGCGTCA
	Reverse: GGAAGCACTGGGGGAAGT
**GAPDH**	Forward: AGCCACATCGCTCAGACAC
	Reverse: GCCCAATACGACCAAATCC

### Western blot analysis

The cells were washed with phosphate-buffered saline and lysed in RIPA buffer (Sigma-Aldrich, St. Louis, MO, USA). The protein lysates were quantified and equal amounts of total protein were separated by sodium dodecyl sulfate-polyacrylamide gel electrophoresis. The separated proteins were transferred from the gel onto polyvinylidene fluoride membranes. Next, the membranes were blocked with 5% skim milk and then incubated with primary antibodies overnight at 4°C. After incubation, the membranes were exposed to secondary antibodies that were conjugated to horseradish peroxidase. Finally, the blots were detected and visualized using enhanced chemiluminescence reagents (Millipore, Burlington, MA, USA). The primary antibodies against the following proteins were used for Western blotting: Dicer (ab14601; Abcam, Cambridge, UK), ADSL (A6278; Abclonal, Woburn, MA, USA), PYCR1 (A13346, Abclonal, Woburn, MA, USA), PRODH (22980-1-AP, Proteintech, Chicago, IL, USA), FTH1 (3998, Cell Signaling, Danvers, MA, USA), and α-tubulin (sc-8035, Santa Cruz Biotechnology, Dallas, TX, USA).

### Plasmid transfection and lentiviral infection

pcDNA6.1-ADSL, pcDNA6.1-Dicer plasmids and pLKO.1-puro-based lentiviral vectors (shDicer and shADSL) were purchased from the National RNAi Core Facility at Academia Sinica, Taipei, Taiwan. An empty pcDNA6.1 plasmid was used as the control. The plasmids were transfected into the cells by using Lipofectamine 2000 (Invitrogen, Waltham, MA, USA) according to the manufacturer’s instructions and incubated for 48 h. To identify cells that had successfully incorporated the plasmids, stably transduced cells were identified by treating the cells with blasticidin. Recombinant lentiviruses were packaged according to the manufacturer’s recommendations. For lentiviral infection, the target cells were incubated with the lentiviral supernatant and 5 μg/mL polybrene for 48 h to establish stable cell lines, which were then selected using puromycin.

### Immunohistochemical staining

Immunohistochemical (IHC) staining was performed using the streptavidin–biotin–peroxidase complex. In brief, melanoma tissue samples were fixed and embedded in paraffin, after which they were dewaxed, rehydrated, and subjected to antigen retrieval. The samples were blocked with a blocking buffer for 30 min at room temperature and then treated with primary (against Dicer and ADSL) and secondary antibodies. Images of the stained tissue sections were captured using a light microscope.

### Bioinformatics

Data on the Dicer and ADSL expression in patients with melanoma were obtained from The Cancer Genome Atlas (TCGA) through the UALCAN data analysis portal (http://ualcan.path.uab.edu/) and analyzed. Subsequently, the Kaplan–Meier melanoma survival analysis was performed on the basis of the differential expression profiles of Dicer and ADSL; for this, we used the PrognoScan database (http://dna00.bio.kyutech.ac.jp/PrognoScan/).

### Statistical analysis

All statistical analyses were performed using GraphPad Prism (version 6). Data from at least three independent experiments were analyzed. The results are presented in terms of the mean ± standard deviation values. The significance of the results was assessed using the Student’s *t* test, and *P* values of < 0.05 were considered to be statistically significant.

## RESULTS

### Inhibition of Dicer expression reduced DTIC sensitivity in melanoma cells

**Figure 1:**
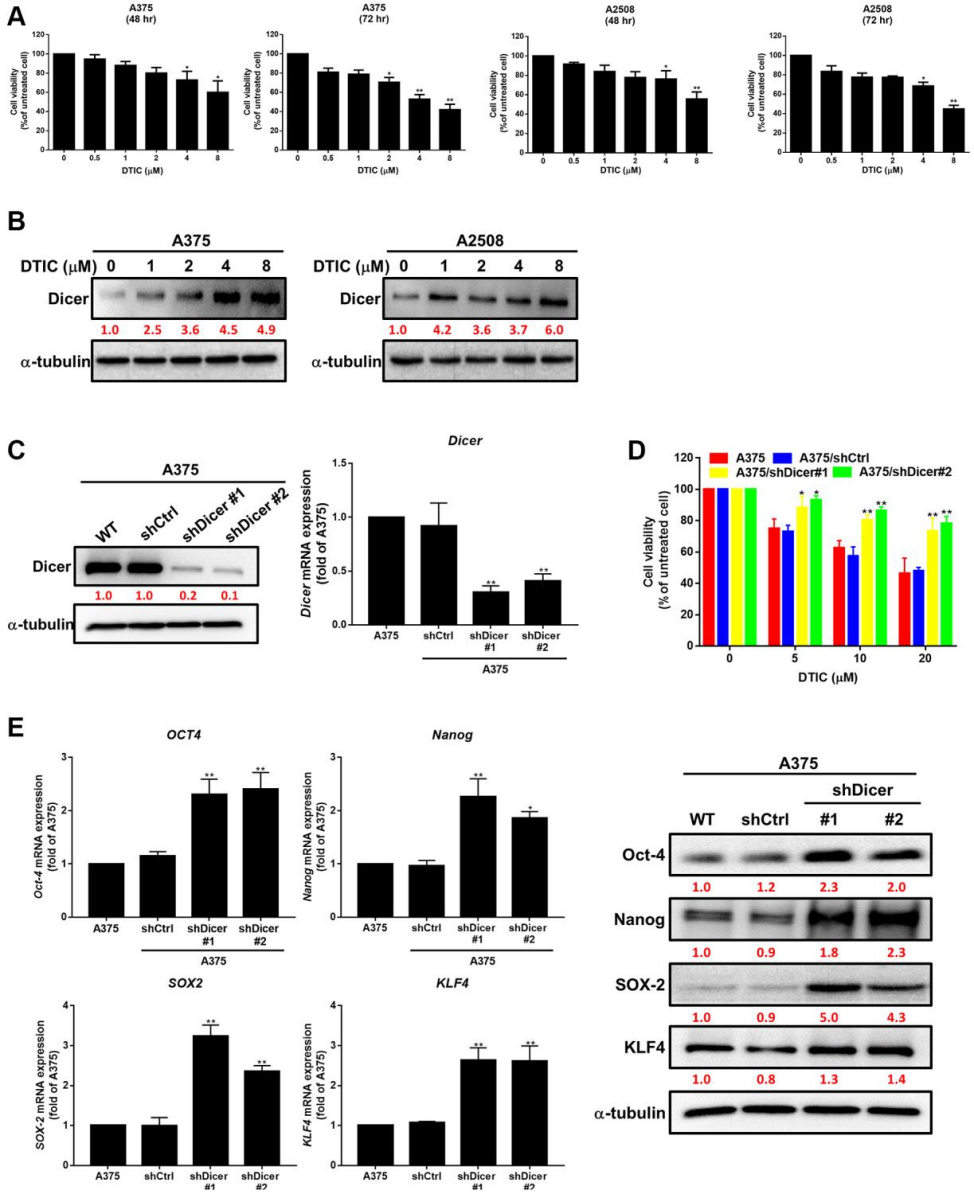
**Association of Dicer expression with dacarbazine (DTIC) treatment response and cancer stemness in melanoma cells.** (**A**) Viability of A375 and A2508 melanoma cells treated with varying concentrations of DTIC for 48 and 72 h. (**B**) Western blotting results indicating the expression levels of Dicer in A375 and A2508 melanoma cells treated with varying concentrations of DTIC. (**C**) Western blotting results indicating the expression levels of Dicer in A375 and Dicer-silenced (shDicer #1 and #2) cells. (**D**) Viability of melanoma cells treated with varying concentrations of DTIC (measured through the MTT assay). (**E**) Expression levels of Oct-4, Nanog, SOX2, and KLF4 in Dicer-silenced (shDicer #1 and #2) cells (evaluated through qRT-PCR and Western blotting). Data are presented in terms of the mean ± standard error of the mean of three independent experiments, each performed in triplicate. ^*^P < 0.05 and ^**^P < 0.01 (Student’s *t* test).

To investigate the therapeutic effects of DTIC on melanoma cells, we treated two melanoma cell lines with varying concentrations of DTIC. We observed that the cell viability of melanoma cells was markedly reduced after treatment with varying concentrations of DTIC for 48 and 72 h (Figure 1A). Moreover, to investigate whether DTIC regulates Dicer signaling in melanoma cells, we evaluated the expression level of Dicer in the DTIC-treated melanoma cells through Western blotting. The results revealed a marked increase in the level of Dicer expression in a concentration-dependent manner (Figure 1B). This finding suggests that Dicer plays a role in modulating the therapeutic effect of DTIC on melanoma cells. We further investigated whether Dicer regulates the therapeutic efficacy of DTIC in melanoma cells. For this purpose, stable Dicer silencing was performed in A375 human melanoma cells (shDicer #1 and #2) by using a lentiviral system; gene silencing was confirmed through Western blotting and qRT-PCR (Figure 1C). We found that Dicer silencing reduced the sensitivity of the melanoma cells to DTIC treatment (Figure 1D). Given the association between reduced chemotherapeutic sensitivity and cancer stemness, we examined the correlations between Dicer expression and stem cell–like characteristics. Our investigations revealed that Dicer silencing was correlated with an increase in the mRNA and protein expression levels of the following cancer stem cell–related transcription factors, as indicated by qRT-PCR and Western blotting (Figure 1E): Oct-4, Nanog, KLF4, and SOX2. These findings collectively indicate that a Dicer plays a pivotal role in modulating DTIC sensitivity and cancer stemness in melanoma cells.

### Dicer expression is associated with the clinical characteristics of patients with melanoma

**Figure 2:**
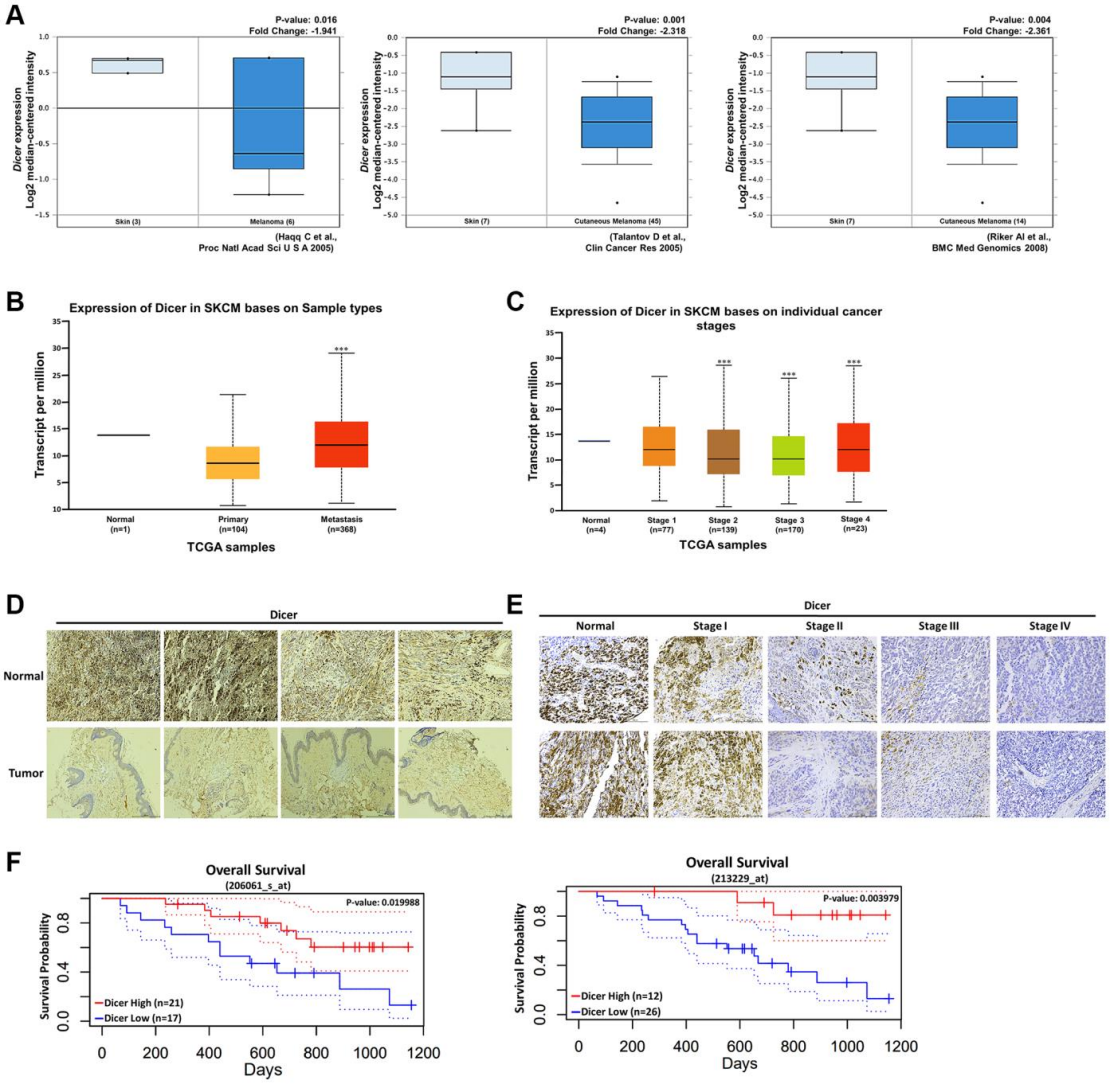
**Clinical relevance of Dicer expression in patients with melanoma.** (**A**) Levels of Dicer expression in melanoma tissues and normal skin tissues, measured using the Oncomine data set. Dicer expression was inversely correlated with (**B**) primary and metastatic melanoma tissues. (**C**) Association between Dicer expression and melanoma tumor stages. (**D**) IHC analysis of the levels of Dicer expression in normal skin tissues and melanoma tissues, (**E**) IHC analysis of the levels of Dicer expression in tissues sampled during various stages of melanoma. Scale bar: 500 μm. (**F**) Kaplan–Meier overall survival curves for the levels of Dicer expression in patients with melanoma.

To investigate the association between Dicer expression and melanoma, we analyzed Dicer expression in patients with melanoma by using data from multiple databases. Dicer expression was markedly downregulated in melanoma tissues compared with the level in normal skin tissues, as revealed by the analysis of data obtained from the Oncomine database (https://www.oncomine.org/resource/login.html; Figure 2A). Furthermore, our analysis of the TCGA data (through the UALCAN analysis portal) highlighted substantial reductions in the levels of Dicer expression in both primary and metastatic melanoma tissues compared with the levels in normal skin tissues (Figure 2B). Additionally, an inverse correlation was observed between Dicer expression and clinical stage in patients with melanoma (Figure 2C). To further corroborate these findings, we performed IHC staining of melanoma tissues; the results confirmed that the level of Dicer expression was considerably lower in melanoma tissues than in normal tissues (Figure 2D). Additionally, Dicer expression was higher in early-stage melanoma tissues and lower in late-stage melanoma tissues (Figure 2E). Data analysis from the PrognoScan database indicated that Dicer expression was prominently associated with overall survival (Figure 2F). These bioinformatic analyses strongly suggest that Dicer is a tumor suppressor whose gene expression correlates with melanoma prognosis.

### Dicer inhibition reduces DTIC sensitivity and enhances cancer stemness through the regulation of ADSL expression

**Figure 3:**
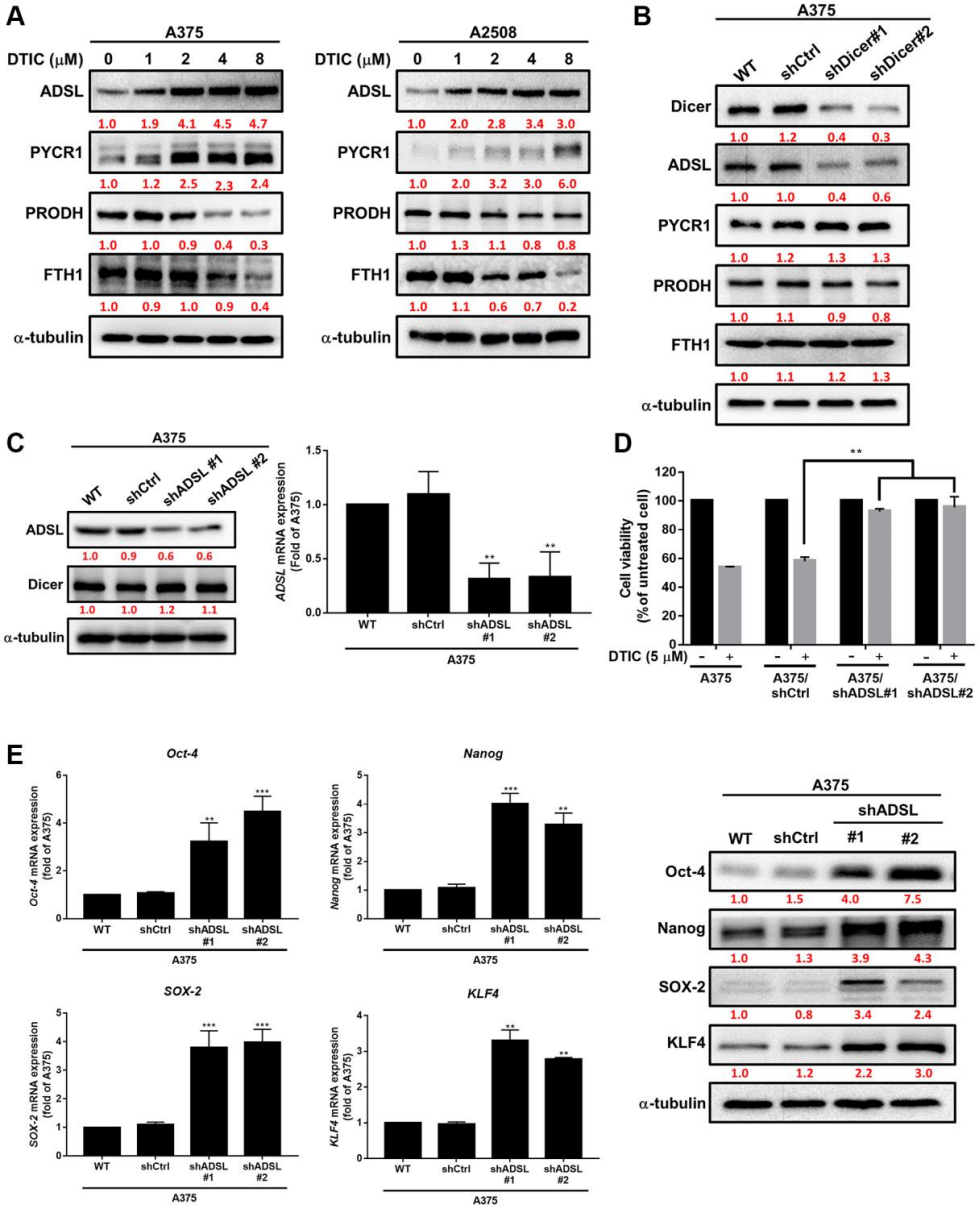
**Association of ADSL expression with DTIC response in melanoma cells.** (**A**) Western blotting results for A375 and A2508 melanoma cells treated with DTIC at the indicated concentrations. (**B**) Western blotting results for metabolic pathway–associated enzymes in wild-type and Dicer-silenced A375 cells. (**C**) Western blotting results indicating ADSL and Dicer levels in wild-type, shctrl, and ADSL-silenced (shADSL #1 and #2) A375 cells. (**D**) MTT assay results indicating the viability of ADSL-silenced A375 cells after incubation with DTIC for 48 h. (**E**) Expression levels of Oct-4, Nanog, SOX2, and KLF4 mRNA (measured through qRT-PCR and Western blotting). ^**^P < 0.01 and ^***^P < 0.001 (Student’s *t* test).

**Figure 4:**
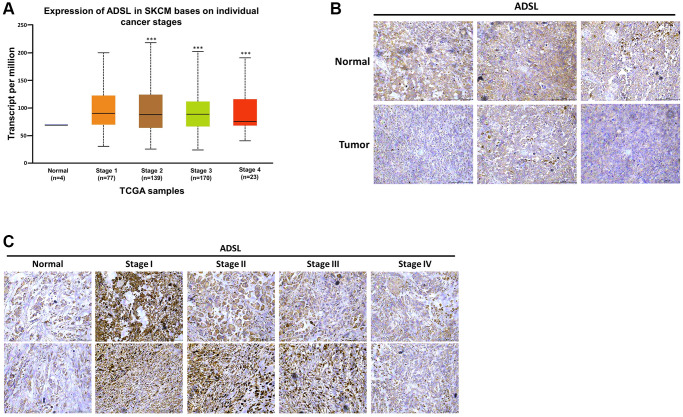
**Clinical implications of ADSL expression in human melanoma.** (**A**) Relationship between ADSL expression and melanoma stage. Representative images of IHC staining for ADSL in (**B**) normal skin tissues and melanoma tissues and (**C**) in tissues sampled during various stages of melanoma. Scale bar: 500 μm.

Metabolic dysregulation has been shown to affect the therapeutic efficacy of drugs [21, 25]. We investigated whether DTIC influenced the expression of metabolic enzymes in melanoma cells. DTIC significantly affected the expression of several metabolic enzymes, including ADSL, as well as enzymes associated with the proline cycle (PYCR1 and PRODH) and iron metabolism (FTH1) (Figure 3A). These findings suggest that DTIC treatment contributes to a metabolic imbalance in melanoma cells. Western blotting was performed to investigate whether Dicer regulates metabolic pathways and modulates the therapeutic effects of DTIC in melanoma cells. Our results indicated that Dicer silencing significantly suppressed the expression of ADSL but not that of the other studied metabolic enzymes (Figure 3B). This observation led us to infer that Dicer induces DTIC resistance through ADSL regulation. Subsequently, we investigated whether ADSL affects the sensitivity of melanoma cells to DTIC. First, we established melanoma cell lines with stable ADSL silencing; gene silencing was confirmed through Western blotting (Figure 3C). Notably, Dicer expression remained unaltered in these cells. Additionally, we examined metabolic enzymes in Dicer-overexpressing A2508 cells and observed that Dicer overexpression upregulated the expression of ADSL but not that of the other studied metabolic enzymes (Supplementary Figure 1). This led us to speculate that Dicer regulates ADSL expression, but ADSL does not regulate Dicer expression. Furthermore, the silencing of ADSL expression was observed to reduce the sensitivity of melanoma cells to DTIC (Figure 3D). ADSL silencing significantly upregulated the mRNA and protein expression levels of cancer stem cell–related transcription factors, namely, Oct-4, Nanog, KLF4, and SOX4, as indicated by qRT-PCR and Western blotting (Figure 3E). We further assessed ADSL expression using patient data obtained from TCGA database and found considerably lower levels of ADSL expression in melanoma tissues than in nonmelanoma tissues (depending on the tumor stage; Figure 4A). IHC staining further confirmed lower ADSL expression levels in melanoma tissues than in normal skin tissues (Figure 4B). Notably, the level of ADSL expression was found to be high in early-stage melanoma tissues and low in late-stage melanoma tissues (Figure 4C). These results collectively highlight the key role of ADSL in influencing DTIC sensitivity and cancer stemness in melanoma cells as well as its potential association with poor melanoma prognosis.

### ADSL signaling is involved in Dicer-mediated DTIC resistance and cancer stem cells properties in melanoma cells

**Figure 5:**
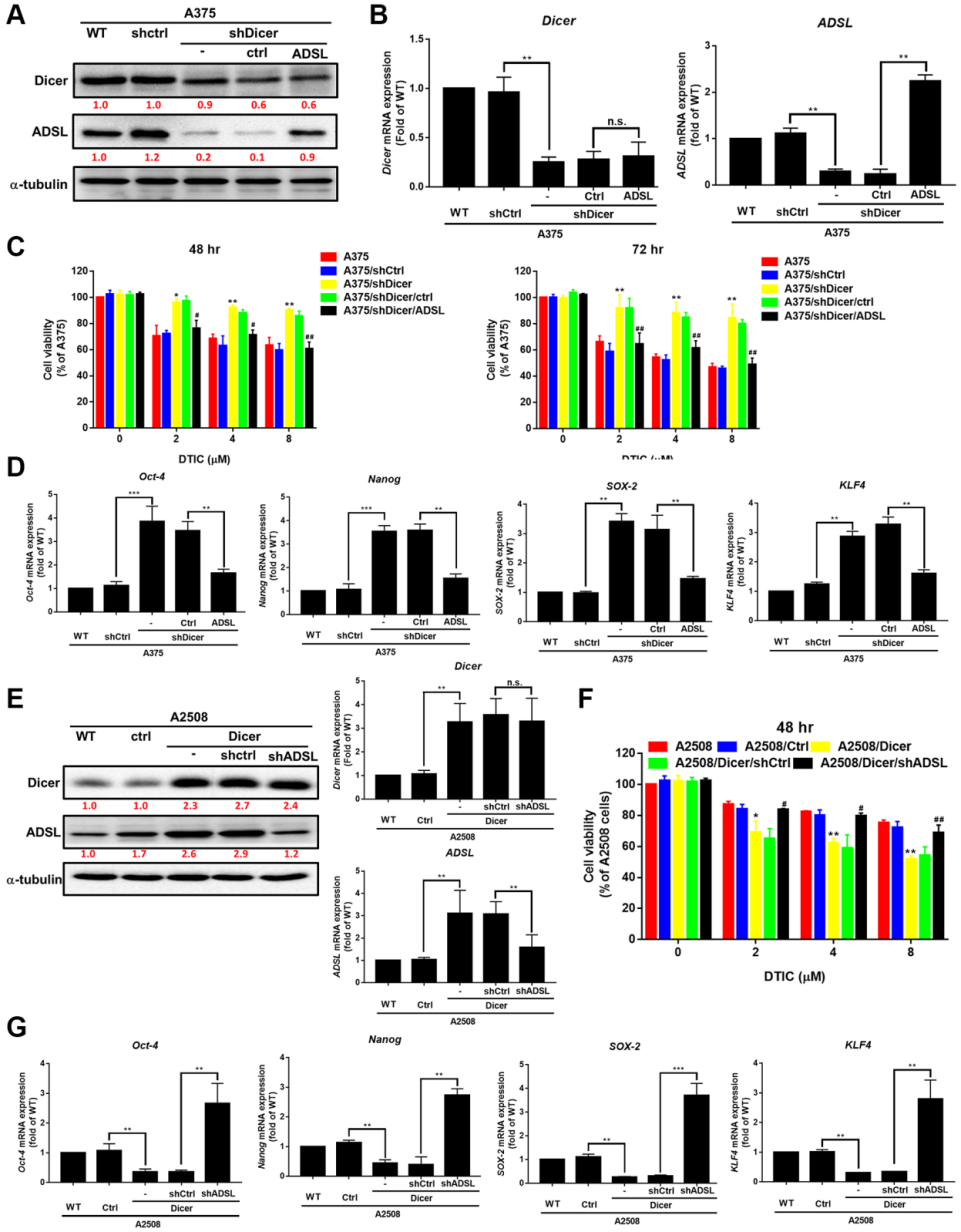
**Role of ADSL signaling in Dicer-mediated DTIC resistance and stemness in melanoma cells.** (**A**) Western blotting results and (**B**) qRT-PCR results indicating the levels of Dicer and ADSL in Dicer-silenced A375 cells transfected with ADSL overexpression plasmids. (**C**) Cell viability (assessed through the MTT assay) 48 and 72 h after treatment with DTIC. (**D**) Expression levels of Oct-4, Nanog, SOX2, and KLF4 mRNA (measured through QRT-PCR). (**E**) Western blotting and qRT-PCR results indicating the levels of Dicer and ADSL in Dicer-overexpressing A2508 cells transfected with ADSL-silenced plasmids. (**F**, **G**) Cell viability (assessed through the MTT assay) and the expression levels of various cancer stem cell markers (measured through qRT-PCR) upon treatment with varying concentrations of DTIC: Oct-4, Nanog, SOX2, and KLF4. Data are presented in terms of the mean ± standard error of the mean of three independent experiments, each performed in triplicate. ^**^P < 0.01 and ^***^P < 0.001 (Student’s *t* test).

**Figure 6:**
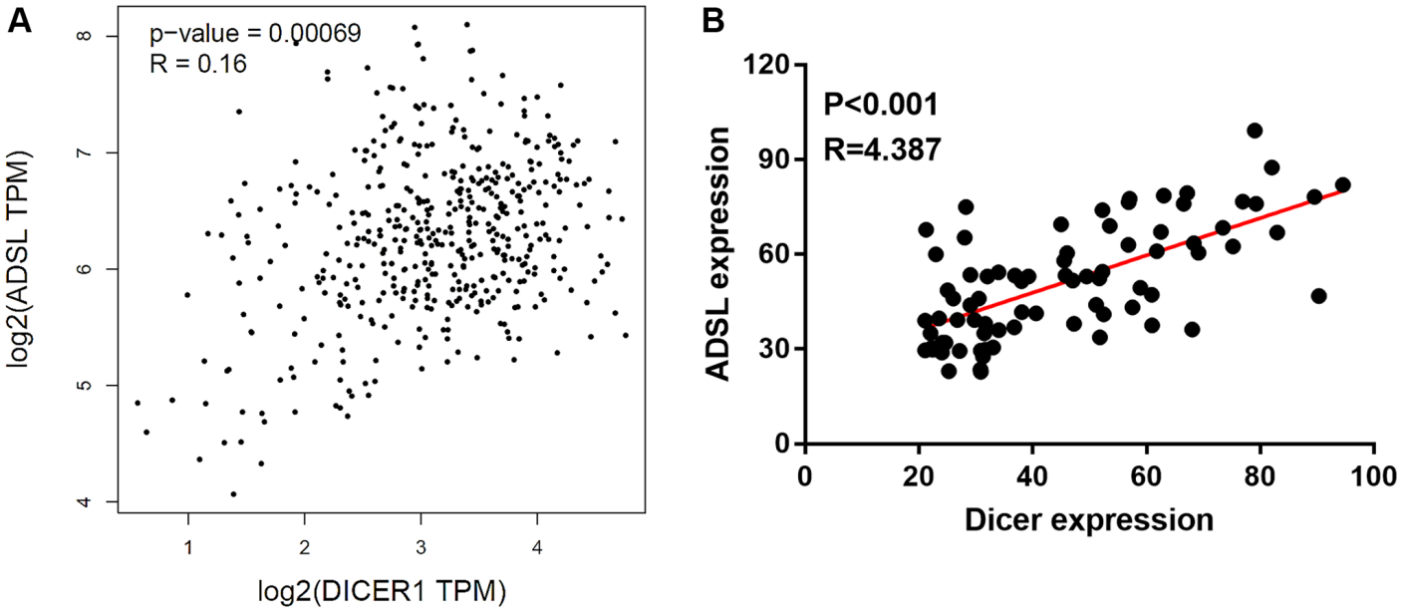
**Clinical relevance of Dicer and ADSL expression in melanoma.** (**A**) Positive correlation observed between Dicer and ADSL levels in patients with melanoma, as evident from the results of TCGA data analysis. (**B**) IHC staining of a melanoma tissue array illustrating the positive association between the expression levels of Dicer and ADSL in melanoma tissue.

Gene manipulation was confirmed through Western blotting. The transfection of the ADSL-expressing vector into Dicer-silenced A375 cells successfully reinstated the expression levels of ADSL protein (Figure 5A) and mRNA (Figure 5B). However, this manipulation did not alter Dicer expression. Moreover, we observed that changes in ADSL levels affected Dicer-mediated DTIC sensitivity (Figure 5C) and cancer stemness (Figure 5D). Conversely, the silencing of ADSL through vector transfection into A2508/Dicer cells (Figure 5E) resulted in the recovery of Dicer-mediated DTIC resistance (Figure 5F) and increases in the levels of stemness markers (Figure 5G). These findings collectively establish ADSL as a crucial downstream mediator of Dicer-regulated DTIC sensitivity and stemness in melanoma cells. The analysis of patient data from TCGA data set further revealed that Dicer expression is positively associated with ADSL expression (Figure 6A); consistent results were observed in the tissue microarrays of patients with melanoma (Figure 6B). These clinical findings indicate that Dicer is strongly correlated with ADSL expression and survival outcomes in melanoma.

## DISCUSSION

Skin cancer is a common malignancy with a poor prognosis. In the United States, skin cancer is the fifth most frequently diagnosed cancer, affecting approximately 20% of the population [26–28]. Skin cancer encompasses various subtypes, such as basal cell carcinoma, squamous cell carcinoma, melanoma, and other rare types [27, 29, 30]. The prevalence of basal cell carcinoma, squamous cell carcinoma, and melanoma is approximately 80%, 20%, and 1%, respectively. Despite its low prevalence, melanoma accounts for approximately 80% of all skin cancer–related mortalities. The substantial effect of melanoma on both individuals and the economy underscores its importance as a public health concern [31, 32]. The primary drug used in the treatment of advanced-stage metastatic melanoma is DTIC. However, the majority of patients develop resistance to DTIC within a few weeks or months of treatment [33, 34]. To enhance the efficacy of DTIC, the genetic regulatory network involved in the effects of DTIC treatment must be elucidated and novel therapeutic targets and diagnostic markers must be identified.

Emerging evidence indicates that Dicer expression exerts tissue-specific effects on various cancers. Dicer is upregulated in colorectal cancer cells and the precursor lesions of lung adenocarcinomas but downregulated in ovarian cancer cells [35–37]. Moreover, studies have revealed that both high and low levels of Dicer expression can be correlated with chemosensitivity in specific patient populations. For example, Dicer knockdown induces significant G1 cell cycle arrest and increases cisplatin sensitivity. Furthermore, Dicer regulates gemcitabine resistance in pancreatic cancer [38], promotes chemotherapeutic response in liver cancer, and contributes to gefitinib resistance in lung cancer [39]. Dicer is also associated with poor chemotherapeutic response in colorectal and ovarian cancers [36, 40]. Consequently, Dicer can serve as a predictor of clinical response. In a study of patients with oral squamous cell carcinoma who received 5-fluorouracil-based chemoradiotherapy, the rate of overall survival was markedly lower among patients with low levels of Dicer expression than among those with high levels of Dicer expression [41]. In a study of patients with HCC, the rate of survival was lower among patients with low levels of Dicer expression than among those with high levels of Dicer expression [42]. The present study revealed that DTIC treatment significantly upregulated Dicer expression in melanoma cells lines in a dose-dependent manner. Additionally, Dicer silencing resulted in a significant reduction in the sensitivity of melanoma cells to DTIC. Furthermore, our clinicopathological analysis revealed that Dicer expression was downregulated in melanoma tissues but not in normal skin tissues. Notably, we found that patients with melanoma who had low levels of Dicer expression exhibited worse survival outcomes than did those with high levels of Dicer expression. These results collectively underscore the major roles played by Dicer in DTIC resistance and stemness in melanoma. Our findings suggest that Dicer might be a novel cancer-suppressing target and prognostic marker in melanoma.

Studies have indicated that the metabolic pathways in cancer cells vary from those in most normal tissue cells [43, 44]. For example, enhanced aerobic glycolysis and generation of biosynthetic intermediates needed for cancer cell differentiation, and cell growth or decreased oxidative phosphorylation [45, 46]. These metabolic alterations not only contribute to tumor development but also mediate the sensitization of tumor cells to antitumor drugs [47, 48]. For example, the upregulation of multidrug resistance protein 2 can induce cisplatin resistance in ovarian cancer cells through the glutathione synthesis pathway [49]. Furthermore, the activity of glucose-6-phosphate dehydrogenase has been associated with the sensitivity of HCC cells to paclitaxel, adriamycin, and cisplatin [50]. Furthermore, sodium/glucose cotransporter 1 overexpression has been shown to promote tamoxifen resistance in breast cancer cells by increasing glycolytic metabolism [51]. However, whether Dicer-mediated alterations in metabolic enzymes contribute to DTIC resistance and cancer stemness in melanoma remains unclear.

We found that DTIC significantly affected the expression levels of various metabolic enzymes, including ADSL, PYCR1, PRODH, and FTH1, in melanoma cells. Dicer silencing suppressed the expression of ADSL but not that of the other metabolic enzymes. Notably, ADSL silencing in A375 cells did not alter Dicer expression. On the basis of these findings, we surmise that Dicer regulates ADSL expression (but ADSL does not regulate Dicer expression), thus influencing DTIC sensitivity in melanoma cells. This prompted us to investigate the role of Dicer-mediated ADSL in the DTIC sensitivity of melanoma cells. ADSL is a crucial enzyme for *de novo* purine biosynthesis. Evidence suggests that alterations in *de novo* purine biosynthesis can affect the stem cell properties of breast cancer cells [52]. Additionally, targeting metabolic enzymes related to this pathway has been shown to alter the expression of stemness-related transcription factors and affect the temozolomide sensitivity of glioblastoma cells [53]. ADSL catalyzes two vital steps in purine biosynthesis: the conversion of succinylaminoimidazole carboxamide ribonucleotide into aminoimidazole carboxamide ribonucleotide and the conversion of adenylosuccinate into adenosine monophosphate. Studies have revealed that ADSL plays a role in prostate cancer progression by inhibiting the cell cycle pathway [54]. Additionally, ADSL contributes to the development of triple-negative breast cancer by activating cMYC [55]. Notably, represses ADSL expression and inhibits the long noncoding RNA MIR22HG to regulate the proliferation and invasion of triple-negative breast cancer cells [56]. These results suggest for the first time that Dicer-mediated ADSL expression is correlated with DTIC resistance and stemness in melanoma.

This study has several limitations that should be acknowledged. First, approximately 40–60% of all patients with melanoma harbor *BRAF* variants. The cell lines A375 and A2508 were selected in this study because of the stability in producing results. However, additional studies using a broader range of cell lines, including primary tumor cell lines with *BRAF* variants, are needed to determine whether Dicer-mediated ADSL expression exerts a similar effect on these cells. Second, Dicer plays a crucial role in the maturation of miRNAs, which regulate gene expression and play essential roles in tumorigenesis, drug resistance, and cancer metastasis and invasion. Numerous studies have revealed that miRNAs regulate cellular metabolism either directly by targeting the key enzymes of metabolic pathways or indirectly by modulating the levels of essential transcription factors involved in metabolism. Therefore, investigating the Dicer-regulated miRNAs implicated in the posttranscriptional regulation of ADSL expression is crucial. Furthermore, we were unable to establish DTIC-resistant melanoma cell lines in this study. In future projects, we plan to develop DTIC-resistant melanoma cell lines and elucidate the mechanisms underlying the regulatory roles of ADSL and Dicer in DTIC resistance.

**Figure 7:**
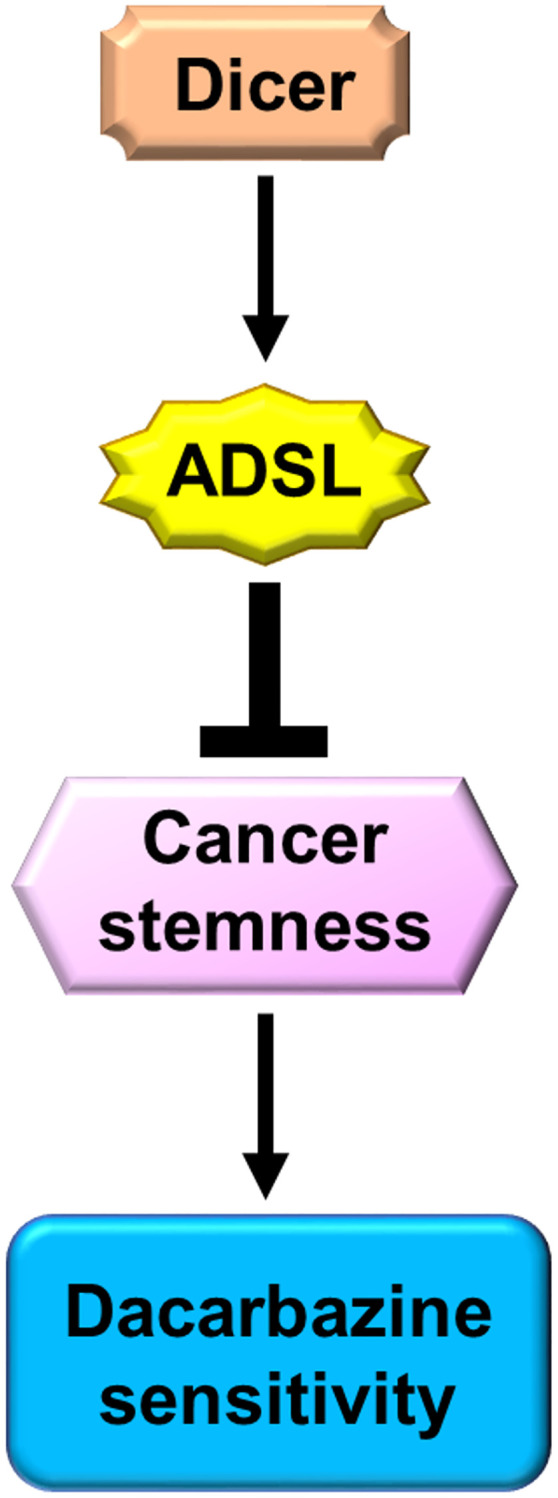
Schematic of Dicer-induced, ADSL-mediated DTIC resistance and augmented stemness in melanoma cells.

Dicer is downregulated in melanoma cells, and a low level of Dicer expression is strongly associated with poor melanoma prognosis. Furthermore, Dicer silencing leads to DTIC resistance and an increase in cancer stemness in melanoma cells by suppressing the downstream expression of ADSL (Figure 7). Our study may serve as a reference for future studies aimed at identifying novel therapeutic targets and biomarkers relevant in melanoma.

## Supplementary Materials

Supplementary Figure 1
