# Supplementary material for: Silencing of Dicer enhances dacarbazine resistance in melanoma cells by inhibiting ADSL expression
Source: Aging (Albany NY). 2023 Nov 15;15(22):12873–89. doi: 10.18632/aging.205207 (PMC10713419; doi:10.18632/aging.205207)
Supplement: Supplementary Figure 1 [file aging-15-205207-s001.pdf]

SUPPLEMENTARY FIGURE

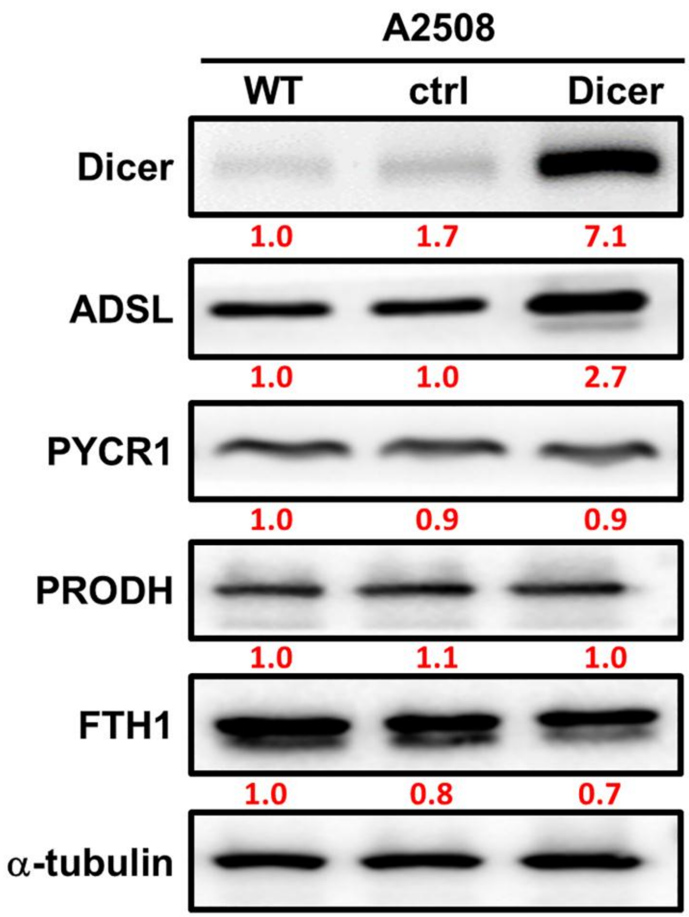

Supplementary Figure 1. Dicer mediated metabolic pathway–associated enzymes in melanoma cells. Western blotting results for metabolic pathway–associated enzymes in wild-type, ctrl, Dicer-overexpressed A2508 cells.
